# Removal of Ni(II), Cu(II), Pb(II), and Cd(II) from Aqueous Phases by Silver Nanoparticles and Magnetic Nanoparticles/Nanocomposites

**DOI:** 10.1021/acsomega.3c04054

**Published:** 2023-09-11

**Authors:** Muradiye ŞAHİN, Muhammet ATASOY, Yasin ARSLAN, Dilek YILDIZ

**Affiliations:** † Kırşehir Ahi Evran University, Campus, Kırşehir 40100, Turkey; ‡ Muğla Vocational School, Chemistry and Chemical Treatment Technologies Department, Chemistry Technology Program, Muğla Sıtkı Koçman University, Muğla 48000, Turkey; § Faculty of Arts and Science, Nanoscience and Nanotechnology Department, Burdur Mehmet Akif Ersoy University, Burdur 15000, Turkey; ∥ Environmental Problems Research and Application Center, Muğla Sıtkı Kocçman University, Muğla 48000, Turkey

## Abstract

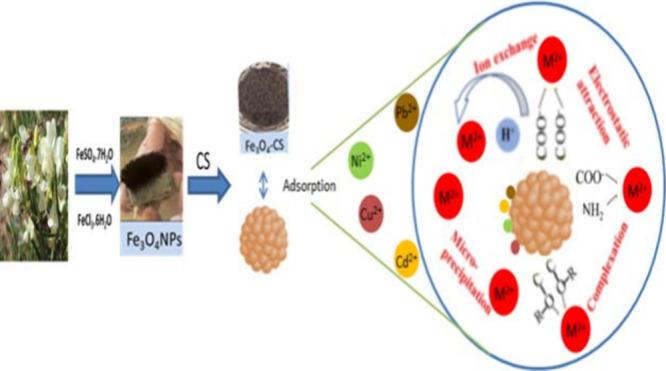

The intake of heavy metals into the body, even at very low concentrations, may cause a decrease in central nervous system functions; deterioration of blood composition; and liver, kidney, and lung damage. Therefore, heavy metal ions must be removed from water. In this study, silver, magnetic iron/copper, and iron oxide nanoparticles were synthesized using *Lathyrus brachypterus* extract and then Fe/Cu-AT, Fe_3_O_4_-AT, Fe/Cu-CS, and Fe_3_O_4_–CS magnetic nanocomposite beads were synthesized using alginate and chitosan. The removal of Cd(II), Pb(II), Ni(II), and Cu(II) ions from aqueous phases using synthesized nanoadsorbents was investigated by single and competitive (double and quaternary) adsorption techniques. The kinetic usability of the magnetic iron oxide chitosan (Fe_3_O_4_–CS) nanocomposite beads with the highest removal efficiency was evaluated. Based on experimental results, the order of removal was found to be 98.39, 75.52, 51.54, and 45.34%, and it was listed as Pb(II) > Cu(II) > Cd(II) > Ni(II), respectively. The Dubinin–Radushkevich, Freundlich, Langmuir, and Temkin isotherm models were used, and experimental results revealed that the experimental data fit the Langmuir model better. The maximum adsorption capacities (*q*_m_) obtained from the Langmuir isotherm model of Fe_3_O_4_–CS were found to be 8.71, 23.75, 18.57, and 12.38 mg/g for Ni(II), Pb(II), Cu(II), and Cd(II) ions, respectively. When the kinetic data were applied to the Lagergren, Ho–McKay, and Elovich models, it was observed that the adsorption kinetics mostly conformed to the Ho–McKay second-order rate equation. The binary and quaternary competitive adsorption data showed that Fe_3_O_4_–CS were selective toward Cu(II) and Pb(II). The reusability of the Fe_3_O_4_–CS nanoadsorbent was performed as three cycles with the same concentration. The adsorption capacities were found to be 95.81, 70.65, 50.50, and 42.75%, in turn for Pb(II), Cu(II), Cd(II), and Ni(II) ions after three cycles, which revealed that the Fe_3_O_4_–CS nanoadsorbent can be used after three cycles without losing its efficiency.

## Introduction

Heavy metal ions are among the most important factors causing environmental pollution. The main cause of heavy metal pollution in waters is the mining industry. The wastes released in the process of obtaining metal from the ore become a source of pollution when undergoing certain processes.^1,2^ These wastes dissolve into the waters with the help of atmospheric effects. Metal ions, which are generally included in the body through the skin or directly through the digestive system and food, show their effects by binding to certain parts of proteins in the cell. Heavy metals can cause vital problems even when taken at low concentrations.^3,4^ Pb, one of the heavy metals, is used as a production aid in many sectors from glass to ammunition and it is especially used in battery and battery production.^5^ Pb is taken into the body by contact, respiration, and feeding ways, and then it mixes with the blood, binds to the erythrocytes, and has a toxic effect. When exposed to high doses, it causes damage to neurological functions.^6−9^ According to the International Agency for Research on Cancer (IARC), Pb is in the class of second-class carcinogens. Cu is widely used in water pipes, valves, roof coatings, inorganic paints, pesticides, nutritional additives, fungicides, and algaecides. In case of excessive exposure, it can cause liver and kidney disorders.^10^ The source of Cu in waters is usually the power and alloy industries, paper mills, and oil refineries.^11^ According to the IARC, Cu is not a carcinogenic metal. Cd has an important area of use in iron, steel, brass, and aluminum plating, especially due to its high resistance to corrosion and stable surface formation. The batteries formed with Ni, Hg, and Ag (Ni–Cd, Hg–Cd, and Ag–Cd batteries) can be counted as the most important usage area of Cd.^12^ Because it is difficult to be eliminated from the body, it can accumulate over time and cause damage to the kidneys, lungs, and liver, even in low amounts.^13^ It can be adsorbed from the intestines and stored in the bones instead of calcium, causing osteoporosis.^14^ Ni is resistant to external influences due to its paramagnetic feature. For this reason, it is used in the electrolytic coating of goods, in obtaining alloys with high wear resistance, and in the production of special steel and coins. It is also used as a catalyst in hydrogenation reactions in batteries, accumulators, paints, glazes, and ceramics to give green color to glass. Ni is generally found in wastewater of industries in which it is used. The most important effect encountered is nickel allergy. In case of long-term exposure to Ni, damage occurs on the skin, kidneys, heart, and lungs as a result of accumulation in the body.^15,16^ The IARC has defined all nickel compounds in the class of carcinogens, except metallic nickel. As a result, heavy metals accumulate in organisms, reach harmful levels, and pose a threat to life. For this reason, the determination and removal of heavy metals in natural waters such as drinking water, lake water, seawater, and wastewater is of great importance in terms of quality of life.

Many methods have been developed and applied to remove heavy metals from water. Among these, adsorption is frequently used to remove heavy metal ions from environments such as water and air.^17−20^ Activated carbon, ion exchange resins, biological materials, various polymeric materials, nanoparticles, alumina, and silica are mostly used as adsorbent in adsorption studies.^21−28^ Due to their unique thermal, electrical, and optical properties, AgNPs are used as sensors for detecting contaminants, such as heavy metals and dyes.^29^ In addition, AgNPs are of great interest in catalysis processes due to their high catalytic effect and antibacterial properties.^30,31^ However, their high cost limits their usage. Magnetic nanoparticles, due to their properties such as large surface area, stable composition, chemical inertness, nontoxicity, structure suitable for modification, resistance to organic solvents, thermal stability, easy availability, low cost, and easy recovery, can be used to release metal ions from aqueous solutions and play an important role in removal efforts.^32−34^ It has been found that bimetallic magnetic nanomaterials (Fe–Ni, Fe–Cu, Fe–Mn, etc.) show higher adsorption capacity for heavy metal than single metal magnetic nanomaterials.^35−37^ However, since the nanoparticles used in wastewater treatment are insoluble, they can cause toxic effects due to their elimination defects.^38^ Therefore, in recent years, nanocomposite adsorbents modified with environmentally friendly, biocompatible polymers, such as chitosan, alginate, and cellulose, have attracted attention in wastewater treatment applications to retain nanoparticles. In addition, polymer-based nanocomposites are ideal for minimizing the agglomeration problem.^37,39,40^

In this study, AgNPs and both magnetic Fe/CuNPs and Fe_3_O_4_NPs were synthesized at room temperature by a green method using the plant extract of *Lathyrus brachypterus* and they were modified with natural polymers, such as alginate and chitosan, to form Fe/Cu-CS, Fe/Cu-AT, Fe_3_O_4_–CS, and Fe_3_O_4_-AT magnetic beads. All the synthesized nanoparticles (metallic, magnetic, and bimetallic) and nanocomposites were used as adsorbent to compare the adsorption of four different heavy metals, namely, Pb(II) Cd(II), Cu(II), and Ni(II), from synthetic waters at different time intervals (30 min, 2 h). The kinetic, isotherm, and reusability studies were performed with Fe_3_O_4_–CS that provided the highest removal efficiency in a shorter time. The synthesized Fe_3_O_4_–CS can be easily dispersed in the environment in which it is applied in adsorption applications and can be collected rapidly with the help of a magnet. Both selectivity and efficiency are very important for the adsorbent during the adsorption of pollutants.^41^ Compared to the adsorbents given in Table 4, Fe_3_O_4_–CS has a higher removal rate in a shorter time with a higher efficiency. The novelty of this study is to compare the efficiency of the seven different synthesized nanoadsorbents in the removal of four different heavy metals by adsorption. Furthermore, the selectivity, reusability, and adsorption mechanisms of the most efficient Fe_3_O_4_–CS nanoadsorbent were investigated in detail.

## 2 Experimental Section

### 2.1 Chemicals and Reagents

Iron(II) sulfate heptahydrate (FeSO_4·_7H_2_O), iron(III) chloride hexahydrate (FeCl_3_.6H_2_O), chitosan, and sodium hydroxide (NaOH) were provided by Sigma-Aldrich. 1000 mg/L of Pb(II), Cd(II), Cu(II), and Ni(II) standard solutions; acetic acid (CH_3_COOH); and sodium alginate (C_6_H_7_O_6_Na) were received from Merck. Copper(II) sulfate pentahydrate (CuSO_4_.5H_2_O) was provided by Indosaw. Silver nitrate (AgNO_3_) and calcium chloride (CaCl_2_) were provided by Fluka. Since the materials used are of high purity, no purification process was performed. All experimental studies were done using double-distilled water (18.2 MΩ cm).

### 2.2 Instrumentation

The morphological and chemical characterizations of the nanoadsorbents were determined by PerkinElmer Fronter model FTIR, Shimadzu UV-1800 (UV–vis), Bruker D8 Advance model X-ray diffraction (XRD) with a Cu Kα radiation source in the 2θ range from 10 to 90°, Carl Zeiss EVO LS 10 scanning electron microscope (SEM), and TEM-120 kV transmission electron microscope (TEM), and Cd(II), Pb(II), Ni(II), and Cu(II) in the aqueous solution were realized with an Agilent 240 model flame atomic absorption spectrometer (FAAS).

### 2.3 Synthesis of Ag, Fe/Cu, and Fe_3_O_4_ NPs

**Figure 1:**
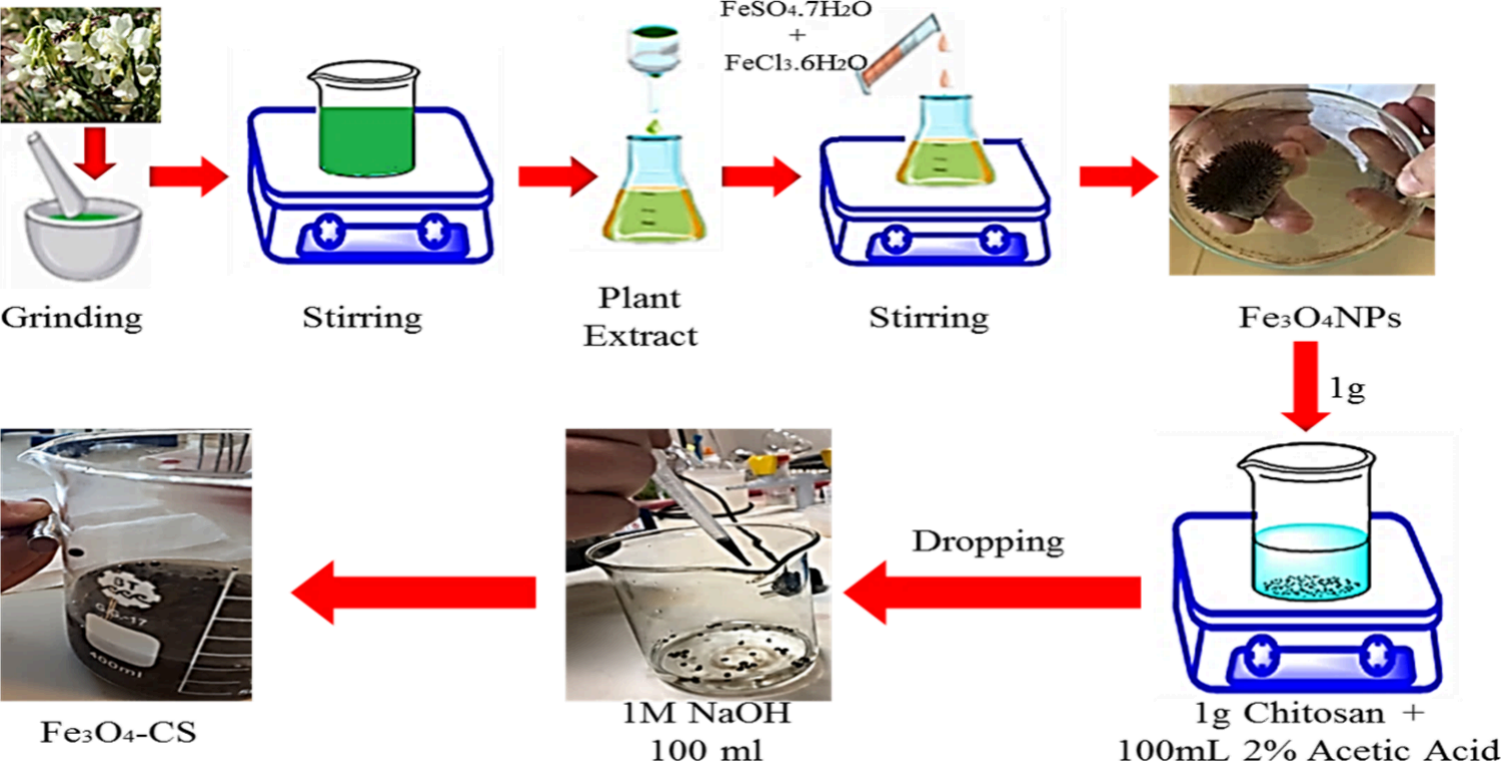
Green synthesis of Fe_3_O_4_NPs and Fe_3_O_4_–CS.

To prepare the extract, 1 g of *Lathyrus brachypterus* was weighed and mixed with 100 mL of distilled water. The mixture was stirred continuously for 5 h at room temperature and afterward separated with a filter paper.^42^ For the synthesis of Fe_3_O_4_NPs, 100 mL of solution including 0.81 g of FeCl_3_·6H_2_O and 0.561 g of FeSO_4_·7H_2_O and 10 mL of plant extract was mixed. To obtain AgNPs, 0.1 M, 45 mL of AgNO_3_ was mixed with 5 mL of extract. To obtain Fe/CuNPs, 100 mL of solution containing 0.18 g of CuSO_4_.5H_2_O and 1.38 g FeSO_4_.7H_2_O was obtained and 10 mL of the plant extract was mixed to this solution.^43,44^ Then, they were mixed at 500 rpm and 25 °C for 30 min on a magnetic stirrer and left to settle (Figure 1). All of the NPs were first separated by a centrifuge and washed three times with distilled water.

While both magnetic Fe_3_O_4_NPs and Fe/CuNPs were separated from the aqueous media with a magnet, AgNPs were separated with a filtrate paper and kept in an oven until usage.^42−44^

### 2.4 Synthesis of Nanocomposite Beads

1.00 g of chitosan was added in 100 mL of solution containing 2% acetic acid, and then stirring was performed until a homogeneous mixture was prepared. Then, 1 g of the magnetic Fe_3_O_4_NPs which we synthesized was added into the mixture and Fe_3_O_4_–CS beads were formed by dropping into 100 mL of 1 M NaOH solution with a dropper. The formed beads were kept for 12 h in NaOH, and then washed with distilled water, and stored at 4 °C in distilled water. The same process was carried out for the synthesis of Fe/Cu-CS by replacing Fe_3_O_4_NPs with Fe/CuNPs.^43,44^ 1.00 g of sodium alginate was added with 100 mL of distilled water, and then stirring was performed until a homogeneous solution was prepared. Then, 0.50 g of Fe/CuNPs was mixed into this solution and sonicated for 30 min. Fe/Cu-AT beads were obtained by dropping into 2% of CaCl_2_ solution with a dropper. The formed beads were washed with distilled water and kept at 4 °C in distilled water. The same process was carried out for the synthesis of Fe_3_O_4_-AT by replacing Fe/CuNPs with Fe_3_O_4_NPs.^43,44^

### 2.5 Adsorption Studies

The removal of heavy metals, such as Cd(II), Pb (II), Ni (II), and Cu (II), from synthetic waters by adsorption on AgNPs, magnetic nanoparticles, and nanocomposites was investigated using the batch adsorption method. 1000 mg/L standard solutions were used. In adsorption experiments, 25 mg of adsorbent was contacted with 25 mL of Pb(II), Cd(II), Cu(II), and Ni(II) solutions separately and four of them together in 100 mL beakers. Adsorption isotherm and kinetic studies were performed at 298 K with an initial heavy metal ion concentration of 10 mg/L in 120 min with Fe_3_O_4_–CS magnetic nanoadsorbents.

The percentage of adsorption removal of Pb(II), Cd(II), Cu(II), and Ni(II)) ions of nanoadsorbents at 298 K was calculated from eq 1:
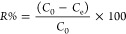
1

*Q*_e_ (mg/g), which is the amount of adsorbed ion, was calculated from eq 2:
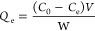
2where *W* is the weight (g) of nanoadsorbent, *V* is the volume of ion solution (L), *C*_e_ is the equilibrium concentrations (mg/L), and *C*_0_ is the initial concentration (mg/L).

## 3 Results and Discussion

### 3.1 Characterization

**Figure 2:**
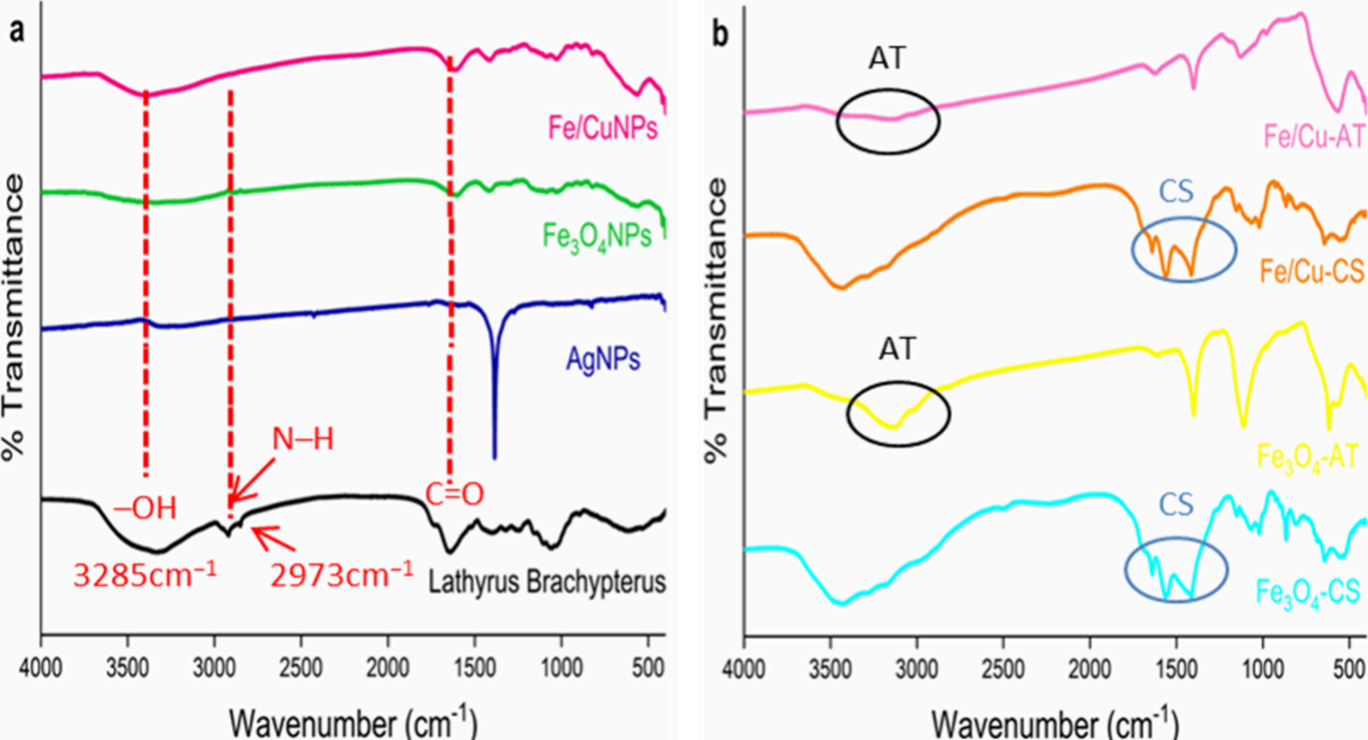
FTIR spectra of (a) extract and NPs. (b) Magnetic nanocomposites

UV–vis spectra of nanoparticles and plant extract are given in Figure S1. The peaks seen in the UV–vis spectra are the characteristic peaks of AgNPs, Fe_3_O_4_NPs, and Fe/CuNPs (Figure S1), and these results showed that all nanoparticles were successfully synthesized. FTIR results of both NPs and plant extract are shown comparatively in Figure 2a. From the FTIR results, it is seen that the plant extract contains carboxyl groups, phenol, amine, alkene, methylene, and alkaline. The fact that most of the signals seen in the plant extract are not seen in the AgNPs suggests that the plant is a good reducing agent with all its functional groups for Ag^+^ to Ag^0^.^42^ FTIR results of Fe/Cu-AT, Fe/Cu-CS, Fe_3_O_4_-AT, and Fe_3_O_4_–CS are given comparatively in Figure 2b. It is confirmed from the FTIR signals that both magnetic nanoparticles were synthesized and coated with chitosan and alginate.^37,43,44^

The result of the XRD analysis performed to define the crystal structure of the synthesized AgNPs is given in Figure S2.^42^ The average particle size of AgNPs found in the calculation using the Scherrer equation with the characteristic Ag (111) peak was found to be 6.08 nm. In the TEM results given in Figure S3, it is seen that AgNPs have a regular crystal structure and do not show great differences in size and form. Besides, it can be given from the size distribution histogram seen in Figure S3 that the nanoparticle size varies between 30 and 6 nm; the mean size of the particles was found to be 14.15 ± 0.20 nm.^42^ The XRD spectra of both magnetic nanoparticles and nanocomposites are shown in Figure S4.^43^ The particle sizes of Fe/CuNPs and Fe_3_O_4_NPs calculated from the peak intensity observed at 2θ = 37.4 and 35.47 in the XRD diffraction model by the Debye–Scherrer equation were calculated to be 18.05 and 11.02 nm, respectively.^43^ The four peaks and six peaks observed in the XRD spectra of Fe/CuNPs and Fe_3_O_4_NPs, respectively, were also observed in the diffraction pattern of both chitosan and alginate nanocomposites, which showed that the coating of both nanocomposites did not change the nanoparticle crystal structure.^43^

**Figure 3:**
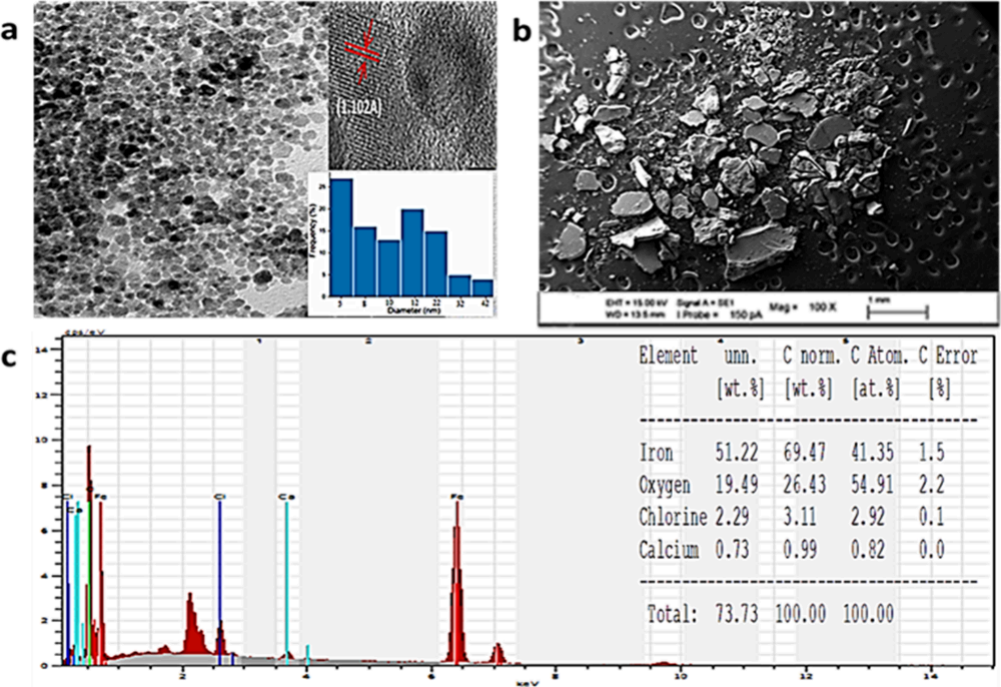
(a) TEM images, (b) SEM images, and (c) EDX analysis of Fe_3_O_4_ NPs.

The size and shape of the Fe_3_O_4_NPs were elucidated by SEM-EDX analysis and TEM images (Figure 3). The SEM images (Figure 3b) are observed for the porous structure of Fe_3_O_4_NPs as well as a series of aggregates. The EDX spectra reveal that both Fe and O atoms are present in the structure of Fe_3_O_4_NPs (Figure 3c). TEM images (Figure 3a) clearly show that the nanoparticles are almost spheric in shape.^37,43^ The size and shape of the Fe/CuNPs were also featured by both SEM and TEM analyses (Figure S5).^43^ The SEM analysis indicates the individual Fe/CuNPs as well as a range of aggregates (Figure S5b). The map data from elemental mapping, consistent with SEM images, confirmed the presence of Cu and Fe (Figure S5b,c).^43^ In the EDX analysis results (Figure S5d), it is seen that the magnetic bimetallic nanoparticle has 35.76% Cu and 58.06% Fe in its structure.^37^Figure S5a indicates the size distribution histogram of the particles, and the average size of the particles is found to be 18.05 ± 0.04 nm; TEM images (Figure S5a) also clearly show that the nanoparticles are almost spheric in shape.^37^

### 3.2 Adsorption Performances of Magnetic Adsorbent in the Aqueous Phase

**Figure 4:**
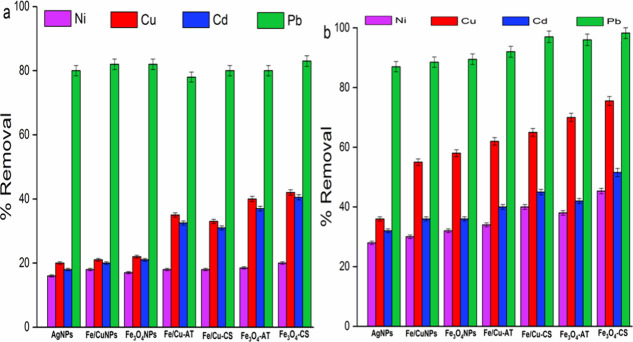
Pb(II), Cd(II), Cu(II), and Ni(II) adsorption of various nanoadsorbents at (a) 30 min and (b) 2 h intervals (initial ion concentration: 10 mg/L, adsorbent dosage: 25 mg/25 mL, *T* = 298 K, RSD% = 2%).

The adsorption of 10 mg/L of 25 mL heavy metal ions (Pb(II), Cd(II), Cu(II), and Ni(II)) on 25 mg AgNPs, magnetic nanoparticles (Fe_3_O_4_NPs and Fe/CuNPs), and nanocomposites (Fe_3_O_4_–CS, Fe/Cu-CS, Fe_3_O_4_-AT, and Fe/Cu-AT) at room temperature in 30 and 120 min was separately investigated, and the removal percentages were calculated using eq 1, as shown in Figure 4. With respect to the experimental results, it was observed that the best adequate adsorbent was found to be Fe_3_O_4_–CS, which adsorbed heavy metals with the highest efficiency at 298 K, as shown in Figure 4. As the particle size decreases, the surface area increases, and as the surface area increases, the amount of adsorbed material also increases. As seen from the TEM and XRD results, Fe_3_O_4_NPs have the smallest particle size and, therefore, the largest surface area. By addition of functional groups with modification, the interaction between adsorbent and adsorbate increases and Fe_3_O_4_–CS is thought to be the best adsorbent depending on the functional group content.

Since Pb(II) was precipitated at pH 6, it was studied at pH 5 for Pb(II) and at pH 6 for other ions. The adsorption reached equilibrium in 120 min. In single adsorption experiments, it was listed as Pb(II) > Cu(II) > Cd(II) > Ni(II) with removals of 98.39, 75.52, 51.54, and 45.34%, respectively. The amount of ions adsorbed on Fe_3_O_4_–CS was determined at fixed wavelengths, such as 217 nm (Pb(II)), 228.8 nm (Cd(II)), 232 nm (Ni(II)), and 324.8 nm (Cu(II)), in the FAAS spectrometer using eq 2 after the magnetic nanoadsorbent was removed from the solutions after a certain period of time by measuring the absorbance of the solution.

**Figure 5:**
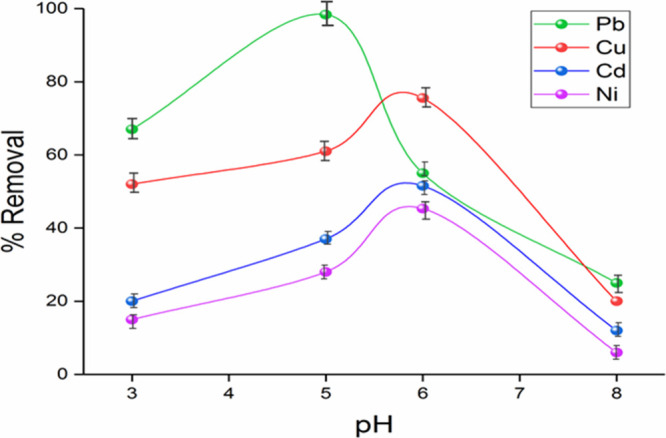
Effect of pH on the removal of Pb(II), Cu(II), Cd(II), and Ni(II) by Fe_3_O_4_–CS (RSD% = 2%).

Since H_3_O^+^ and OH^–^ ions in the solution are also adsorbed on the adsorbent surface, pH is an important factor affecting the adsorption. In addition, temperature, amount of adsorbent, and initial heavy metal concentration parameters also affect the adsorption; however, in this study, since it was aimed to remove traces of heavy metals in wastewater in the easiest and most economical way, only pH optimization was made without optimizing the specified parameters (Figure 5).

As seen in Figure 5, an increase in adsorption was observed with an increase in pH for all heavy metals from pH 3 to 6. It is thought that the low Fe_3_O_4_–CS adsorption efficiency in the acidic environment is due to both the presence of more H^+^ at low pH values and the reduction of the regions that will hold the metal ions in the Fe_3_O_4_–CS bead. In acidic medium, Pb^2+^, Ni^2+^, Cu^2+^, Cd^2+^, and Ni(OH)^+^ are more dominant species in aqueous solution. After pH 6, it started to decrease due to the interaction of the hydroxide group with heavy metals and the activation of the electrostatic force (repulsion) between the hydroxide groups in Fe_3_O_4_–CS. It is observed that metal salts were precipitated as their hydroxyl precipitated at higher pH values.

**Figure 6:**
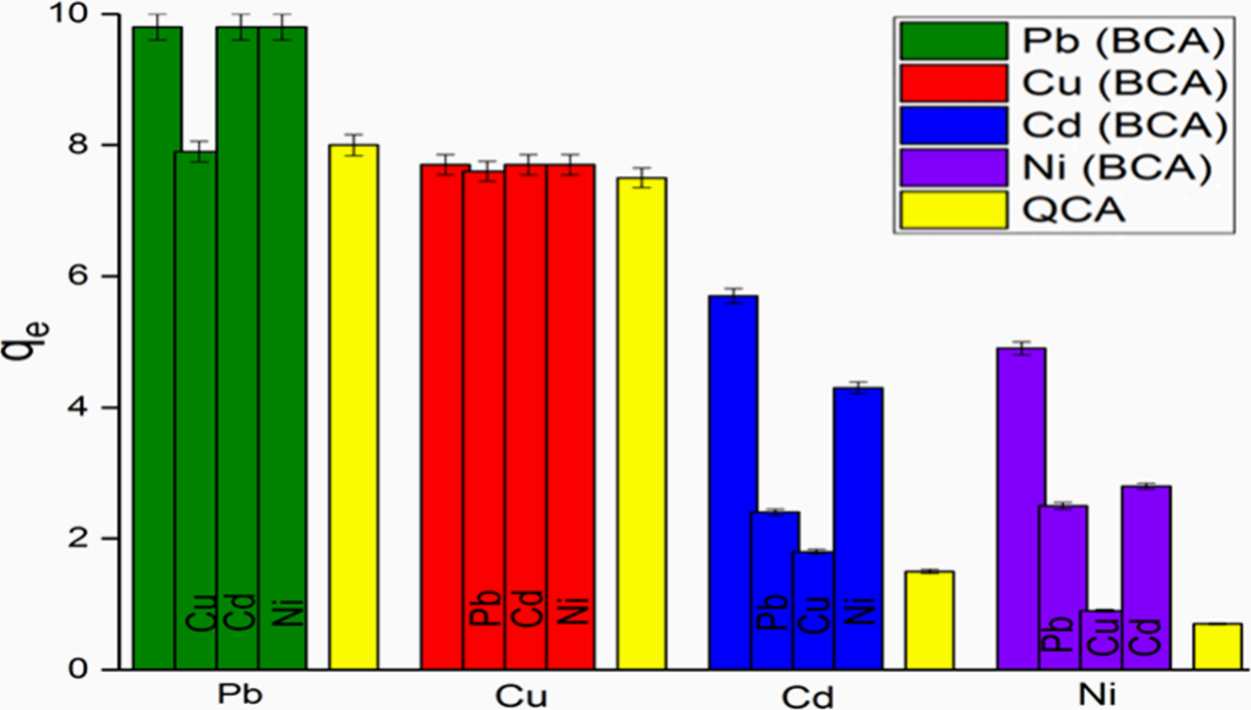
Comparison of Pb(II), Cu(II), Cd(II), and Ni(II) binary and quadruple competitive adsorption capacities (initial ion concentration: 10 mg/L, adsorbent dosage: 25 mg/25 mL, *T* = 298 K, RSD% = 2%).

In principle, environmental wastewater is quite complex. For this reason, it is important to determine the adsorption behavior of more metal ions on Fe_3_O_4_–CS in environments. For this purpose, double and quadruple competitive adsorption experiments were performed and the change in adsorption capacity for each metal ion studied was compared with the case when the metal ion was alone (Figure 6). In competitive adsorption, the concentration of each metal ion was 10 mg/L, with 25 mg Fe_3_O_4_–CS in a sample volume of 25 mL at room temperature for 60 min_._ It is observed that equilibrium was reached in 30 min in competitive adsorption. As seen in Figure 6, in the binary competitive adsorption experiments, it is observed that Fe_3_O_4_–CS adsorbed Cu(II) metal ions at almost the same rate in the presence of other metal ions; that is, it is determined that other metal ions had no interference effect and acted selectively against Cu(II) ions. The same is true for Pb(II) ions, but only Cu(II) ions have an interference effect on Pb(II) adsorption. For Cd(II) and Ni(II) ions, the adsorption performance of Fe_3_O_4_–CS is affected by all other metal ions in the environment and they are the least adsorbed in the presence of Cu(II) ions. In line with the results obtained by double and quaternary competitive adsorption studies, it is revealed that Fe_3_O_4_–CS behaved more selectively toward Cu(II) and Pb(II) ions.

### 3.3 Adsorption Isotherms

**Figure 7:**
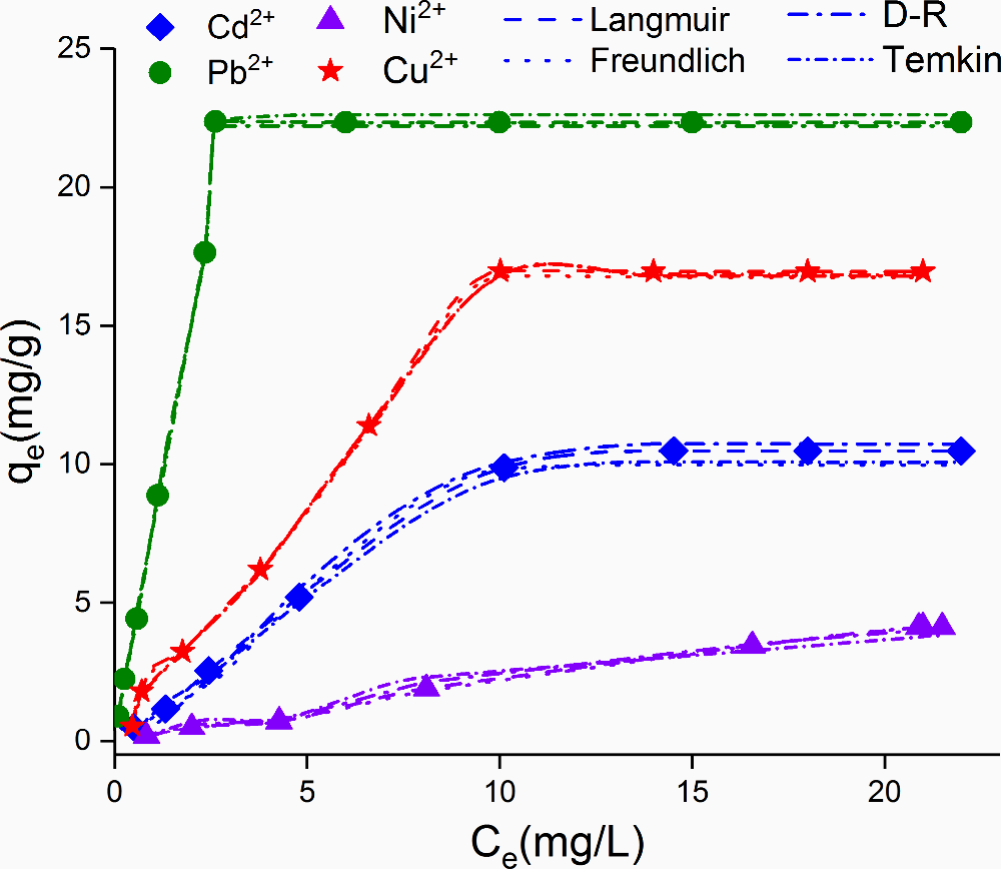
Adsorption isotherm models of Pb(II), Cu(II), Ni(II), and Cd(II) onto Fe_3_O_4_–CS (adsorption conditions: 25 mg/25 mL Fe_3_O_4_–CS, initial metal ion concentration 2–100 mg/L, 120 min) .

The interactions between Fe_3_O_4_–CS and the target metal ions and the properties of the adsorption process on the surface of the particles were tried to be explained by Dubinin–Radushkevich (D–R), Freundlich, Langmuir, and Temkin isotherms (Figure 7). The basic equations of these isotherm models are given in Table 1.

**Table 1:** Isotherm and Kinetic Models Used in the Mathematical Models of Metal Ion Adsorption by Fe_3_O_4_–CS

**isotherm/kinetic models**	**equations**
Langmuir	q_e_ = qmKLCe1+KLCe (3)
Freundlich	q_e_ = KFCe1/n (4)
Dubinin–Radushkevich	q_e_ = qm(−KDRε2) (5)
Temkin	qe = Bln(KTCe) (6)
pseudo-first-order	qt = qe(1—e–k1t) (7)
pseudo-second-order	qt = qe2k2t1+qek2t (8)
Elovich	qt = 1βln(t+αβ) (9)

where *K*_L_ (L/mg) is the Langmuir adsorption constant, *C*_e_ (mg/L) is the equilibrium concentration of the adsorbate, *C*_o_ (mg/L) is the initial dye concentration, *q*_m_ (mg/g) is the theoretical maximum adsorption capacity, and *q*_e_ (mg/g) is the amount of adsorbate adsorbed per unit mass of adsorbent. *K*_F_ ((mg/g) × (mg/L)^*n*^) and *n* are Freundlich constants. Here, *n* is an indicator of how favorable the adsorption process is while *K*_F_ represents the amount of adsorbate adsorbed on an adsorbent per unit equilibrium concentration. The 1/*n* slope, which ranges from 0 and 1, represents a measure of adsorption intensity or surface heterogeneity and becomes more heterogeneous as its value approaches zero.^45^ While *K*_T_ (L/mg) is the equilibrium binding constant, *B* (J/mol) is the Temkin constant based on the heat of adsorption. A negative value of *B* indicates that the process is endothermic, and a positive *B* value indicates that the process is exothermic.^46^*K*_DR_ is the Dubinin–Radushkevizch constant, and ε (kJ/mol) is the adsorption energy.

Since the *R*^2^ value of the line drawn according to the Langmuir model of metal ion adsorption by Fe_3_O_4_–CS at room temperature is closer to 1.00 than the *R*^2^ value drawn according to the Freundlich model (Table 2), it is seen that the adsorption equilibrium model is more suitable for the Langmuir model.

**Table 2:** Langmuir, Freundlich, Temkin, and D–R isotherm Modal Constants for the Adsorption of Pb(II), Cu(II), Ni(II), and Cd(II) on Fe_3_O_4_–CS

**isotherm parameters**		**Pb(II)**	**Cd(II)**	**Cu(II)**	**Ni(II)**
Langmuir isotherm	*q*_m_ (mg/g)	23.75	12.38	18.57	8.71
	*K*_L_ (L/mg)	0.0043	0.0027	0.0031	0.0003
	*R*^2^	0.9983	0.9969	0.9966	0.9918
Freundlich isotherm	*K*_F_ ((mg/g)×(mg/L)^*n*^)	1.134	1.994	1.976	4.787
	*n*	1.038	1.051	1.194	1.009
	*R*^2^	0.9974	0.9879	0.9903	0.9887
Temkin isotherm	*B* (J/mol)	–6.6579	–1.414	–1.531	–0.832
	*K*_T_ (L/mg)	0.398	0.759	0.922	2.013
	*R*^2^	0.9095	0.8364	0.8719	0.8093
Dubinin–Radushkevizch (D–R) isotherm	*q*_m_ (mg/g)	13.335	4.9998	5.759	1.9919
	*K*_DR_ (mol/kJ)^2^	0.0846	0.3003	0.3669	0.6915
	ε (kJ/mol)	2.4331	1.2903	1.1669	0.8503
	*R*^2^	0.8571	0.7300	0.7533	0.7221

### 3.4 Adsorption Kinetics

In order to explain the mechanisms of adsorbed pollutants during the adsorption process, Lagergren (pseudo-first-order kinetics) (PFO, eq 7), Ho–McKay (pseudo-second-order kinetics) (PSO, eq 8), and Elovich kinetic models (eq 9) were applied.

**Figure 8:**
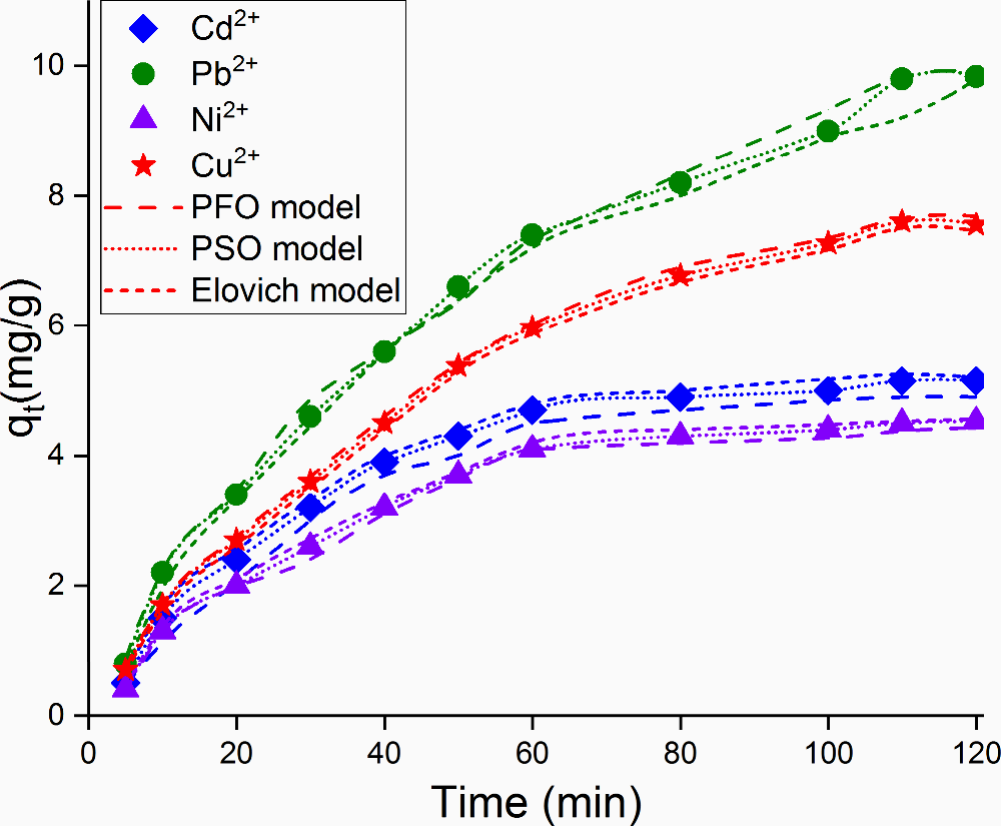
Kinetic models for adsorption of Pb(II), Cu(II), Ni(II), and Cd(II) onto Fe_3_O_4_–CS.

A *t* versus *q_t_* graph is shown in Figure 8 drawn according to three kinetic models at different time intervals of heavy metal ions at a concentration of 10 mg/L adsorbed on Fe_3_O_4_–CS magnetic nanoadsorbent at 298 K. The kinetic parameters calculated with the data obtained from this graph are given in Table 3.

**Table 3:** Modeling the Experimental Data of Pb(II), Cu(II), Ni(II), and Cd(II) Adsorption on Fe_3_O_4_–CS

**kinetic model parameters**		**Pb(II)**	**Cd(II)**	**Cu(II)**	**Ni(II)**
PFO	*q*_e_ (mg/g)	8.927	4.054	6.894	3.271
	*k*_1_ × 10^–3^ (1/min)	4.740	5.273	4.941	5.996
	*R*^2^	0.9784	0.9669	0.9706	0.9658
PSO	*q*_e_ (mg/g)	9.839	5.154	7.552	4.534
	*k*_2_ × 10^–3^ (g/mg·min)	0.0387	0.0051	0.0094	0.0039
	*R*^2^	0.9915	0.9822	0.9879	0.9829
Elovich	β (mg/g)	0.030	0.014	0.031	0.012
	α (g/mg·min)	1.938	1.797	1.902	1.513
	*R*^2^	0.9625	0.9764	0.9819	0.9763

**Figure 9:**
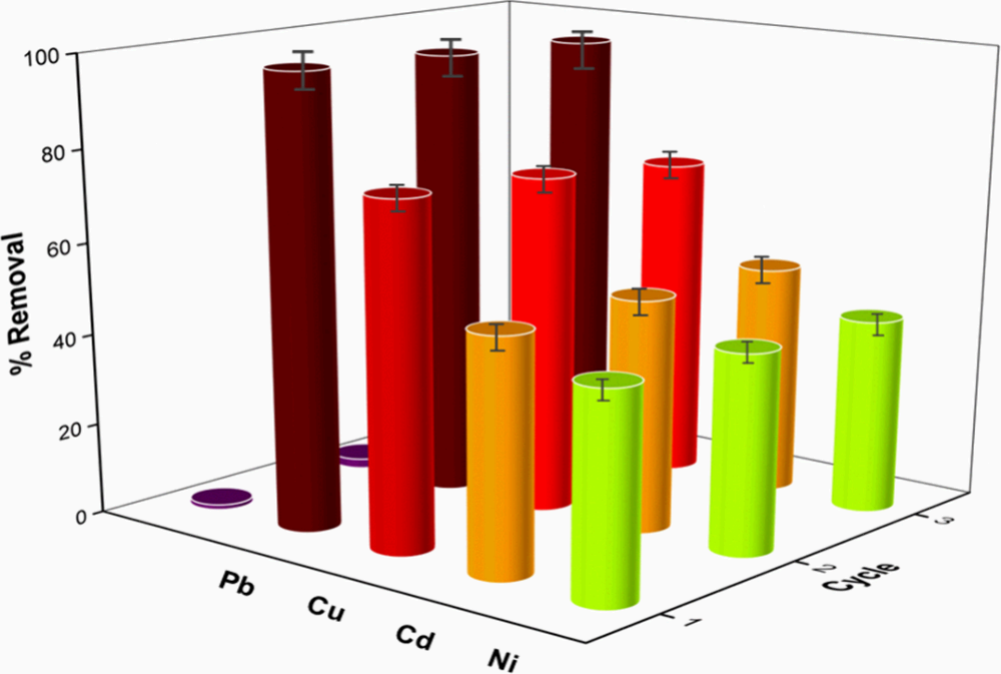
Effect of cycles on the adsorption/desorption capacity for Pb(II), Ni(II), Cd(II), and Cu(II) (RSD% = 2%).

According to the data in Table 3, as the correlation value is closest to 1 for pseudo-second-order kinetics, the Fe_3_O_4_–CS nanoadsorbent is more suitable for the pseudo-second-order kinetic model. In order to investigate the reusability of Fe_3_O_4_–CS adsorbent, adsorption/desorption studies were repeated three times and the results are given in Figure 9. As seen in Figure 9, the adsorbent has a stable and active structure and there is a slight decrease in adsorption capacities.

**Figure 10:**
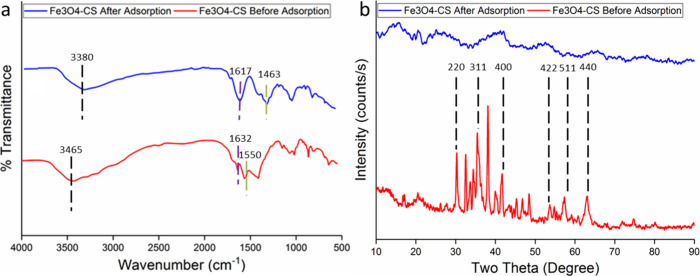
(a) FTIR spectra and (b) XRD models before and after adsorption of Pb(II), Ni(II), Cd(II), and Cu(II) with Fe_3_O_4_–CS.

Adsorption mechanisms include complexation, ion exchange, precipitation, π–π interaction, and the extensive effect of electrostatic species.^47,48^ In order to elucidate the adsorption mechanism, the results of FTIR and XRD analyses before and after adsorption are given in Figure 10.

**Figure 11:**
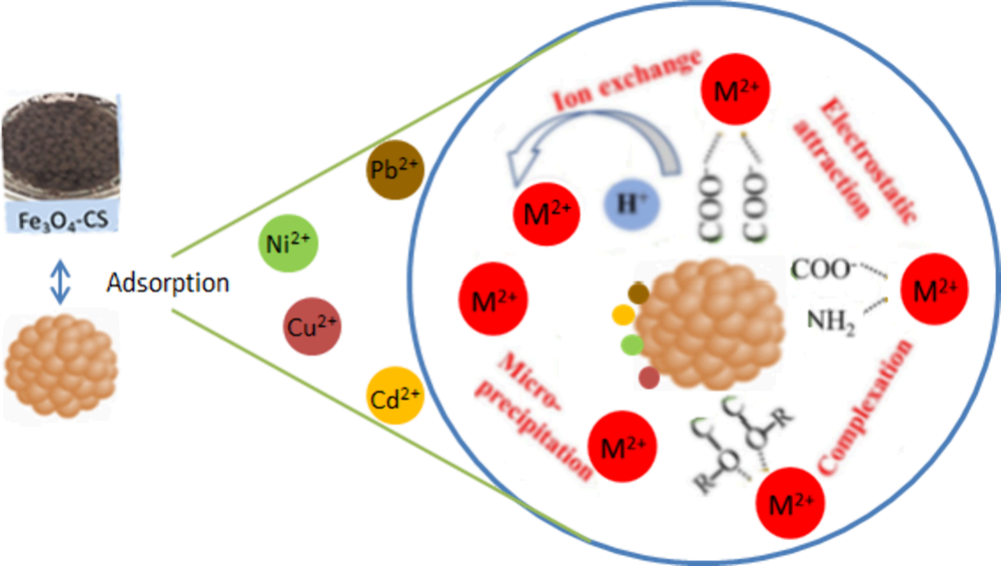
Mechanisms of Pb(II), Ni(II), Cd(II), and Cu(II) adsorption by Fe_3_O_4_–CS.

When the FTIR spectra are compared (Figure 10a), it is seen that the O–H group absorption bond seen at 3500 cm^–1^ shifts to a lower wavenumber and narrows after heavy metal adsorption. It is seen that the asymmetric and symmetrical peaks of −COO in the range 1550–1640 cm^–1^ have also shifted. In this situation, metal ions and functional groups of adsorbent can cause electrostatic attraction and complexation between them. The fact that six peaks belonging to Fe_3_O_4_–CS in the XRD spectra are also seen in the XRD results after adsorption (Figure 10b) indicates that the crystal structure has not deteriorated, and the decrease in all peak intensity values indicates that the adsorption process has taken place. According to the results of the kinetic and isotherm experiments, chemical adsorption on the monolayer surface played an effective role in the removal of Pb(II), Cd(II), Ni(II), and Cu(II); chemical interaction is thought to be the main factor, and the possible physical/chemical interaction mechanism is given in Figure 11.

A comparison of this work with some literature studies on heavy metal removal is given in Table 4.

**Table 4:** A Comparison of the Removal Efficiency of the Prepared Adsorbent with Those Announced in the Literature

adsorbent	Pb (R %) (mg/g)	Cr (R %)	Cu (R %)	Ni (R %)	Hg (R %)	contact time (min)	reference
modified lignin	95.8%					240	(49)
Ni/Fe NPs		90%				150	(50)
Fe_3_O_4_ NPs		99%				120	(51)
P-BNMR@Fe_3_O_4_	92%					240	(52)
Fe/Cu-CS	99%					120	(37)
EDTA- Fe_3_O_4_	68%		70%		62%	Pb: 40 Cu: 90 Hg: 50	(53)
Ppp@Fe_3_O_4_		97%		89%		Cr: 60 Ni: 150	(54)
*b*-cyclodextrin-functionalized biochars	131.24 mg/g					120	(55)
kaolinite nanotubes (KNTs)	89 mg/g	91.6				Pb: 360Cr: 120	(56)
Mg0.5Cu0.5Fe_2_O_4_	57.7 mg/g					120	(57)
Fe_3_O_4_–CS	98.39%		75.52%	45.34%		120	This Work

## 4 Conclusions

The AgNPs and both magnetic Fe_3_O_4_NPs and Fe/CuNPs were synthesized using an easy, environmentally friendly, and economically green method using an endemic plant extract. Then, the magnetic nanoparticles were modified with alginate and chitosan to be used as adsorbent. The structure of the synthesized nanoadsorbents was characterized by FTIR, XRD, SEM-EDX, and TEM methods. The removal efficiencies of 10 mg/L Pb, Ni, Cd, and Cu were investigated by using 25 mg adsorbents at room temperature in both 30 and 120 min, and it was seen that Fe_3_O_4_–CS gave the highest removal efficiency. For this reason, kinetic and isotherm studies were performed with Fe_3_O_4_–CS, which was determined to be the most suitable adsorbent. The removal of Pb(II), Cd(II), Ni(II), and Cu(II) on Fe_3_O_4_–CS adsorbent (single, double, and quadruple competitive) in synthetic waters was investigated in detail, and the optimum conditions were determined for the removal with the highest efficiency in the shortest time. In single adsorption experiments, it was listed as Pb(II) > Cu(II) > Cd(II) > Ni(II) with removals of 98.39, 75.52, 51.54, and 45.34%, respectively. In line with the results obtained by double and quaternary competitive adsorption studies, the single adsorption capacities (*q*_e_) for Pb, Cu, Cd, and Ni are found to be 9.839, 7.552, 5.154, and 4.534, respectively, while the double competitive adsorption capacities for Pb are found to be 7.956, 9.825, and 9.827 in the case of Pb–Cu, Pb–Cd, and Pb–Ni, respectively. The adsorption capacities for Cu are found to be 7.389, 7.550, and 7.548 in the case of Cu–Pb, Cu–Cd, and Cu–Ni, respectively. Furthermore, the adsorption capacities for Cd and Ni were found to be 2.324, 1.183, 4.539, 2.038, 0.565, and 2.703 in the case of Cd–Pb, Cd–Cu, Cd–Ni, Ni–Pb, Ni–Cu, and Ni–Cd, respectively. Moreover, quadruple competitive adsorption capacities were found to be 7.909, 7.448, 0.956, and 0.168 for Pb, Cu, Cd, and Ni, respectively. These results revealed that Fe_3_O_4_–CS behaved more selectively toward both Cu(II) and Pb(II) ions. The mechanism was elucidated by kinetic and isotherm studies. Based on experimental results, while the adsorption kinetics is more suitable for the pseudo-second-order kinetic model, the adsorption isotherm is more suitable for the Langmuir model.

## Supporting Information Available

UV–vis spectra for all NPs, XRD analysis for all compounds, and TEM/SEM images for Fe/CuNPs (PDF)

## References

[ref1] Li X. A.; Zhou D. M.; Xu J. J.; Chen H. Y. In-channel indirect amperometric dedection of heavy metal ions for electrophoresis on a poly(dimethylsiloxane) microchip. Talanta 2000, 71, 1130–1135. 10.1016/j.talanta.2006.06.009. 19071423

[ref2] Elçi L.; Kartal A. A.; Soylak M. Solid phase extraction method for the determination of iron, lead and chromium by atomic absorption spectrometry using Amberite XAD-2000 column in various water samples. J. Hazard Mater. 2008, 153, 454–461. 10.1016/j.jhazmat.2007.08.075. 17928136

[ref3] García-Rosales G.; Colín-Cruz A. Biosorption of lead by maize (Zea mays) stalk sponge. J. Environ. Manage. 2010, 91 (11), 2079–2086. 10.1016/j.jenvman.2010.06.004. 20615602

[ref4] Bulut Y.; Baysal Z. Removal of Pb(II) from wastewater using wheat bran. J. Environ. Manage. 2006, 78 (2), 107–113. 10.1016/j.jenvman.2005.03.010. 16046250

[ref5] Mishra P. C.; Patel R. K. Removel of lead and zinc ions from water by low cost adsorbents. J. Hazard Mater. 2009, 168, 319–325. 10.1016/j.jhazmat.2009.02.026. 19299083

[ref6] Sun D. T.; Peng L.; Reeder W. S.; Moosavi S. M.; Tiana D.; Britt D. K.; Oveisi E.; Queen W. L. Rapid, selective heavy metal removal from water by a metal-Organic framework/polydopamine composite. ACS Cent Sci. 2018, 4, 349–356. 10.1021/acscentsci.7b00605. 29632880 PMC5879484

[ref7] Rakhym A. B.; Seilkhanova G. A.; Kurmanbayeva T. S. Adsorption of lead (II) ions from water solutions with natural zeolite and chamotte clay. Mater. Today: Proc. 2020, 31, 482–485. 10.1016/j.matpr.2020.05.672.

[ref8] Saravanan A.; Kumar P. S.; Yaashikaa P. R.; Karishma S.; Jeevanantham S.; Swetha S. Mixed biosorbent of agro waste and bacterial biomass for the separation of Pb (II) ions from water system. Chemosphere 2021, 277, 130236 10.1016/j.chemosphere.2021.130236. 33770696

[ref9] Atasoy M. Development of a New Sensitive Method for Lead Determination by Platinum-Coated Tungsten-Coil Hydride Generation Atomic Absorption Spectrometry. ACS Omega 2023, 8, 22866–22875. 10.1021/acsomega.3c01856. 37396250 PMC10308594

[ref10] Georgopoulos P. G.; Roy A.; Yonone-Lioy M. J.; Opiekun R. E.; Lioy P. J. Environmental copper: its dynamics and human exposure issues. J. Toxicol Environ. Health Part B 2001, 4, 341–394. 10.1080/109374001753146207. 11695043

[ref11] Tapiero H.; Townsend D. M.; Tew K. D. Trace elements in human physiology and pathology. Copper. Biomed. Pharmacother. 2003, 57, 386–387. 10.1016/S0753-3322(03)00012-X. 14652164 PMC6361146

[ref12] Järup L.; Åkesson A. Current status of cadmium as an environmental health problem. Toxicol. Appl. Pharmacol. 2009, 238, 201–208. 10.1016/j.taap.2009.04.020. 19409405

[ref13] Higashikawa F. S.; Conz R. F.; Colzato M.; Cerri C. E. P.; Alleoni L. R. F. Effects of feedstock type and slow pyrolysis temperature in the production of biochars on the removal of cadmium and nickel from water. J. Clean. Prod. 2016, 137, 965–972. 10.1016/j.jclepro.2016.07.205.

[ref14] Viaene M. K.; Masschelein R.; Leenders J.; De Groof M.; Swerts L. J.; Roels H. A. Neurobehavioural effects of occupational exposure to cadmium: a cross sectional epidemiological study. Occup. Environ. Med. 2000, 57 (1), 19–27. 10.1136/oem.57.1.19. 10711265 PMC1739855

[ref15] Denkhaus E.; Salnikow K. Nickel Essentiality, Toxicity and Carcinogenicity. Crit Rev. Oncol/Hematol. 2002, 42, 35–56. 10.1016/S1040-8428(01)00214-1. 11923067

[ref16] Kasprzak K. S.; Sunderman F. W. Jr; Salnikow K. Nickel Carcinogenesis. Mutat. Res., Fundam. Mol. Mech. Mutagen. 2003, 533, 67–97. 10.1016/j.mrfmmm.2003.08.021. 14643413

[ref17] Xu C.; Fang R.; Luque R.; Chen L.; Li Y. Functional metal–organic frameworks for catalytic applications. Coord. Chem. Rev. 2019, 388, 268–292. 10.1016/j.ccr.2019.03.005.

[ref18] Zhang P.; Zhang X.; Li Y.; Han L. Influence of pyrolysis temperature on chemical speciation, leaching ability, and environmental risk of heavy metals in biochar derived from cow manure. Bioresour. Technol. 2020, 302, 122850 10.1016/j.biortech.2020.122850. 32007849

[ref19] Wang Y.; Yan J.; Wang J.; Zhang X.; Wei L.; Du Y.; Yu B.; Ye S. Superhydrophobic metal organic framework doped polycarbonate porous monolith for efficient selective removal oil from water. Chemosphere 2020, 260, 127583 10.1016/j.chemosphere.2020.127583. 32698115

[ref20] Liu J.; Li G.; Yu Y.; Zhou X. Hierarchically porous covalent organic framework for adsorption and removal of triphenylmethane dyes. Microporous Mesoporous Mater. 2021, 312, 110703 10.1016/j.micromeso.2020.110703.

[ref21] Shahrashoub M.; Bakhtiari S. The efficiency of activated carbon/magnetite nanoparticles composites in copper removal: Industrial waste recovery, green synthesis, characterization, and adsorption-desorption studies. Microporous Mesoporous Mater. 2021, 311, 110692 10.1016/j.micromeso.2020.110692.

[ref22] Hayati B.; Maleki A.; Najafi F.; Gharibi F.; McKay G.; Gupta V. K.; Harikaranahalli Puttaiah S.; Marzban N. Heavy metal adsorption using PAMAM/CNT nanocomposite from aqueous solution in batch and continuous fixed bed systems. Chem. Eng. J. 2018, 346, 258–270. 10.1016/j.cej.2018.03.172.

[ref23] Lebron Y. A. R.; Moreira V. R.; Drumond G. P.; da Silva M. M.; Bernardes R. d. O.; Santos L. V. d. S.; Jacob R. S.; Viana M. M.; de Vasconcelos C. K. B. Graphene oxide for efficient treatment of real contaminated water by mining tailings: Metal adsorption studies to Paraopeba river and risk assessment. Chem. Eng. J. Adv. 2020, 2, 100017 10.1016/j.ceja.2020.100017.

[ref24] El-Dib F. I.; Mohamed D. E.; El-Shamy O. A. A.; Mishrif M. R. Study the adsorption properties of magnetite nanoparticles in the presence of different synthesized surfactants for heavy metal ions removal. Egypt J. Pet. 2020, 29, 1–7. 10.1016/j.ejpe.2019.08.004.

[ref25] Peng H.; Gao P.; Chu G.; Pan B.; Peng J.; Xing B. Enhanced adsorption of Cu(II) and Cd(II) by phosphoric acid-modified biochars. Environ. Pollut. 2017, 229, 846–853. 10.1016/j.envpol.2017.07.004. 28779896

[ref26] Adenuga A. A.; Amos O. D.; Oyekunle J. A. O.; Umukoro E. H. Adsorption performance and mechanism of a low-cost biosorbent from spent seedcake of Calophyllum inophyllum in simultaneous cleanup of potentially toxic metals from industrial wastewater. J. Environ. Chem. Eng. 2019, 7, 103317 10.1016/j.jece.2019.103317.

[ref27] Khalfa L.; Sdiri A.; Bagane M.; Cervera M. L. A calcined clay fixed bed adsorption studies for the removal of heavy metals from aqueous solutions. J. Clean Prod. 2021, 278, 123935 10.1016/j.jclepro.2020.123935.

[ref28] Hao W.; Xu J.; Li R.; Zhao X.; Qiu L.; Yang W. Developing superhydrophobic rock wool for high-viscosity oil/water separation. Chem. Eng. J. 2019, 368, 837–846. 10.1016/j.cej.2019.02.161.

[ref29] Daoudi K.; Gaidi M.; Columbus S.; Shameer M.; Alawadhi H. Highly sensitive silver decorated-graphene oxide-silicon nanowires hybrid SERS sensors for trace level detection of environmental pollutants. arXiv preprint arXiv 2021, 2101, 07955 10.48550/arXiv.2101.07955.

[ref30] Şahin M.; Gübbük İ.H. Green synthesis of antioxidant silver and platinum nanoparticles using ginger and turmeric extracts and investigation of their catalytic activity. J. Turkish Chem. Soc. A 2019, 6 (3), 403–410. 10.18596/jotcsa.497440.

[ref31] Chen H.; Gao F.; He R.; Cui D. Chemiluminescence of luminol catalyzed by silver nanoparticles. J. Colloid Interface Sci. 2007, 315, 158–163. 10.1016/j.jcis.2007.06.052. 17681516

[ref32] Gautam R. K.; Gautam P. K.; Banerjee S.; Soni S.; Singh S. K.; Chattopadhyaya M. C. Removal of Ni(II) by magnetic nanoparticles. J. Mol. Liq. 2015, 204, 60–69. 10.1016/j.molliq.2015.01.038.

[ref33] Fato F. T.; Li D.-W.; Zhao L.-J.; Qiu K.; Long Y.-T. Simultaneous removal of multiple heavy metal ions from river water using ultrafine mesoporous magnetite nanoparticles. ACS Omega 2019, 4, 7543–7549. 10.1021/acsomega.9b00731. 31459847 PMC6648574

[ref34] Wang K.; Fu J.; Wang S.; Gao M.; Zhu J.; Wang Z.; Xu Q. Polydopamine-coated magnetic nanochains as efficient dye adsorbent with good recyclability and magnetic separability. J. Colloid Interface Sci. 2018, 516, 263–273. 10.1016/j.jcis.2018.01.067. 29408113

[ref35] Babaee Y.; Mulligan C. N.; Rahaman M. S. Removal of arsenic (III) and arsenic (V) from aqueous solutions through adsorption by Fe/Cu nanoparticles. J. Chem. Biotechnol. 2018, 93, 63–71. 10.1002/jctb.5320.

[ref36] Li Z.; Deng S.; Yu G.; Huang J.; Lim V. C. As(V) and As(III) removal from water by a Ce–Ti oxide adsorbent: Behavior and mechanism. Chem. Eng. J. 2010, 161, 106–113. 10.1016/j.cej.2010.04.039.

[ref37] Şahin M.; Arslan Y. Green synthesis of metal nanoparticles and magnetic nanocomposites for adsorption, desorption and preconcentration of Pb(II). ChemistrySelect 2023, 8, 18 10.1002/slct.202300708.

[ref38] Zhou X. H.; Huang B. C.; Zhou T.; Liu Y. C.; Shi H. C. Aggregation behavior of engineered nanoparticles and their impact on activated sludge in wastewater treatment. Chemosphere 2015, 119, 568–576. 10.1016/j.chemosphere.2014.07.037. 25127355

[ref39] Shi L.; Zhang X.; Chen Z. Removal of chromium (VI) from wastewater using bentonite-supported nanoscale zero-valent iron. Water Res. 2011, 45, 886–892. 10.1016/j.watres.2010.09.025. 20950833

[ref40] Pandey N.; Shukla S. K.; Singh N. B. Zinc oxide-urea formaldehyde nanocomposite film as low-cost adsorbent for removal of Cu(II) from aqueous solution. Adv. Mater. Lett. 2015, 6, 172–178. 10.5185/amlett.2014.5604.

[ref41] Li X.; Cui Y. Y.; Chen Y. J.; Yang C. X.; Yan X. P. Facile synthesis of dual-functionalized microporous organic network for efficient removal of cationic dyes from water. Microporous Mesoporous Mater. 2020, 296, 110013 10.1016/j.micromeso.2020.110013.

[ref42] Şahin M.; Arslan Y.; Tomul F.; Yıldırım B.; Genç H. Green synthesis of silver nanoparticles using Lathyrus brachypterus extract for efficient catalytic reduction of methylene blue, methyl orange, methyl red and investigation of a kinetic model. React. Kinet. Mech Catal. 2022a, 135, 3303–3315. 10.1007/s11144-022-02299-3.

[ref43] Şahin M.; Arslan Y.; Tomul F. Removal of naproxen and diclofenac using magnetic nanoparticles/nanocomposites. Res. Chem. Intermed. 2022b, 48, 5209–5226. 10.1007/s11164-022-04862-y.

[ref44] Şahin M.; Arslan Y.; Tomul F. Adsorption, oxidation, kinetic and thermodynamic studies of methyl orange by magnetic Fe3O4 NPs and their chitosan/alginate nanocomposites. Int. J. Environ. Anal. Chem. 2022c 1-20 10.1080/03067319.2022.2140417.

[ref45] Fytianos K.; Voudrias E.; Kokkalis E. Sorption-desorption behaviour of 2,4-dichlorophenol by marine sediments. Chemosphere 2000, 40 (1), 3–6. 10.1016/S0045-6535(99)00214-3. 10665437

[ref46] Paluri P.; Ahmad K. A.; Durbha K. S. Importance of estimation of optimum isotherm model parameters for adsorption of methylene blue onto biomass derived activated carbons: Comparison between linear and non-linear methods. Biomass Convers. Biorefin. 2022, 12, 4031–4048. 10.1007/s13399-020-00867-y.

[ref47] Yang G. X.; Jiang H. Amino modification of biochar for enhanced adsorption of copper ions from synthetic wastewater. Water Res. 2014, 48, 396–405. 10.1016/j.watres.2013.09.050. 24183556

[ref48] Jin H.; Hanif M. U.; Capareda S.; Chang Z.; Huang H.; Ai Y. Copper(II) removal potential from aqueous solution by pyrolysis biochar derived from anaerobically digested algae-dairy-manure and effect of KOH activation. J. Environ. Chem. Eng. 2016, 4 (1), 365–372. 10.1016/j.jece.2015.11.022.

[ref49] Demirbaş A. Adsorption of lead and cadamium ions in aqueous solutions into modified lignin from alkali glycerol delignieation. J. Hazard. Mater. 2004, 109, 221–226. 10.1016/j.jhazmat.2004.04.002. 15177762

[ref50] Zhou S.; Li Y.; Chen J.; Liu Z.; Wang Z.; Na P. Enhanced Cr( vi ) removal from aqueous solutions using Ni/Fe bimetallic nanoparticles: characterization, kinetics and mechanism. RSC Adv. 2014, 4 (92), 50699–50707. 10.1039/C4RA08754B.

[ref51] Chatterjee S.; Mahanty S.; Das P.; Chaudhuri P.; Das S. Biofabrication of iron oxide nanoparticles using manglicolous fungus Aspergillus niger BSC-1 and removal of Cr(VI) from aqueous solution. Chem. Eng. J. 2020, 385, 123790 10.1016/j.cej.2019.123790.

[ref52] Song T.; Yu C.; He X.; Lin J.; Liu Z.; Yang X.; Zhang Y.; Huang Y.; Tang C. Synthesis of magnetically separable porous BN microrods@Fe3O4 nanocomposites for Pb(II) adsorption. Colloids Surf. A 2018, 537, 508–515. 10.1016/j.colsurfa.2017.10.060.

[ref53] Cui L.; Wang Y.; Gao L.; Hu L.; Yan L.; Wei Q.; Du B. EDTA functionalized magnetic graphene oxide for removal of Pb(II), Hg(II) and Cu(II) in water treatment: Adsorption mechanism and separation property. Chem. Eng. J. 2015, 281, 1–10. 10.1016/j.cej.2015.06.043.

[ref54] Chithra K.; Akshayaraj R. T.; Pandian K. Polypyrrole-Protected Magnetic Nanoparticles as an Excellent Sorbent for Effective Removal of Cr(VI) and Ni(II) from Effluent Water: Kinetic Studies and Error Analysis. Arab J. Sci. Eng. 2018, 43, 6219–6228. 10.1007/s13369-018-3421-x.

[ref55] Zhao H. T.; Ma S. A.; Zheng S. Y.; Han S. E.; Yao F. X.; Wang X. Z.; Wang S. S.; Feng K. β-cyclodextrin functionalized biochars as novel sorbents for high-performance of Pb^2+^ removal. J. Hazard. Mater. 2019, 362, 206–213. 10.1016/j.jhazmat.2018.09.027. 30240994

[ref56] Abukhadra M. R.; Bakry B. M.; Adlii A.; Yakout S. M.; El-Zaidy M. E. Facile conversion of Kaolinite into clay nanotubes (KNTs) of enhanced adsorption properties for toxic heavy metals (Zn^2+,^ Cd^2+^, Pb^2+^, and Cr^6+^) from water. J. Hazard Mater. 2019, 374, 296–308. 10.1016/j.jhazmat.2019.04.047. 31009894

[ref57] Tran C. V.; Quang D. V.; Nguyen Thi H. P.; Truong T. N.; La D. D. Effective Removal of Pb(II) from Aqueous Media by a New Design of Cu–Mg Binary Ferrite. ACS Omega 2020, 5, 7298–7306. 10.1021/acsomega.9b04126. 32280871 PMC7144175

