# Supplementary material for: Removal of Ni(II), Cu(II), Pb(II), and Cd(II) from Aqueous Phases by Silver Nanoparticles and Magnetic Nanoparticles/Nanocomposites
Source: ACS Omega. 2023 Sep 11;8(38):34834–43. doi: 10.1021/acsomega.3c04054 (PMC10536035; doi:10.1021/acsomega.3c04054)
Supplement: Supplementary file 1 — ao3c04054_si_001.pdf [file ao3c04054_si_001.pdf]

Supporting Information

Removal of Ni(II), Cu(II), Pb(II) and Cd(II) from Aqueous Phases by Silver Nanoparticles and Magnetic Nanoparticles/Nanocomposites

Muradiye ŞAHİN [a], Muhammet ATASOY [b,*], Yasin ARSLAN [c], Dilek YILDIZ [d]

[a] Kırşehir Ahi Evran University, Campus, 40100 Kırşehir, Turkey.

[b] Muğla Sıtkı Koçman University, Muğla Vocational School, Chemistry and Chemical Treatment Technologies Department, Chemistry Technology Program, 48000 Muğla, Turkey.

[c] Burdur Mehmet Akif Ersoy University, Faculty of Arts and Science, Nanoscience and Nanotechnology Department, 15000 Burdur, Turkey.

[d] Muğla Sıtkı Koçman University, Environmental Problems Research and Application Center, 48000 Muğla, Turkey.

Corresponding Author : Muhammet ATASOY
E-mail: muhammetkarabas@mu.edu.tr

This provides further information about characterization of AgNPs[1], magnetic nanoparticles and nanocomposites[2]. This material is available free of charge via the internet.

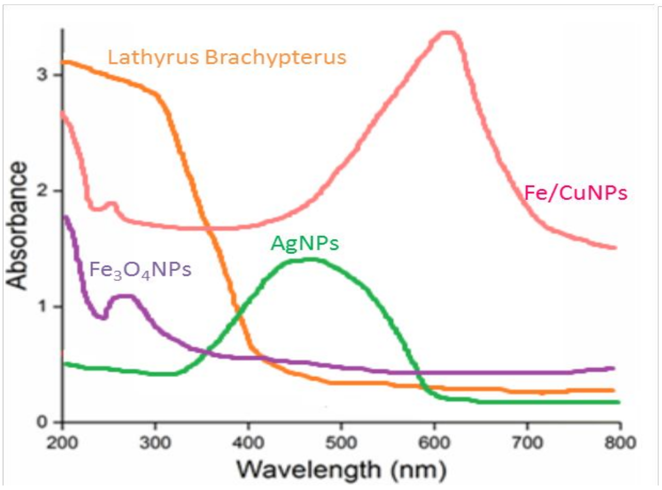

Figure S1. UV–Vis spectra of L. brachypterus extract, AgNPs, Fe/CuNPs and Fe_3O_4NPs.

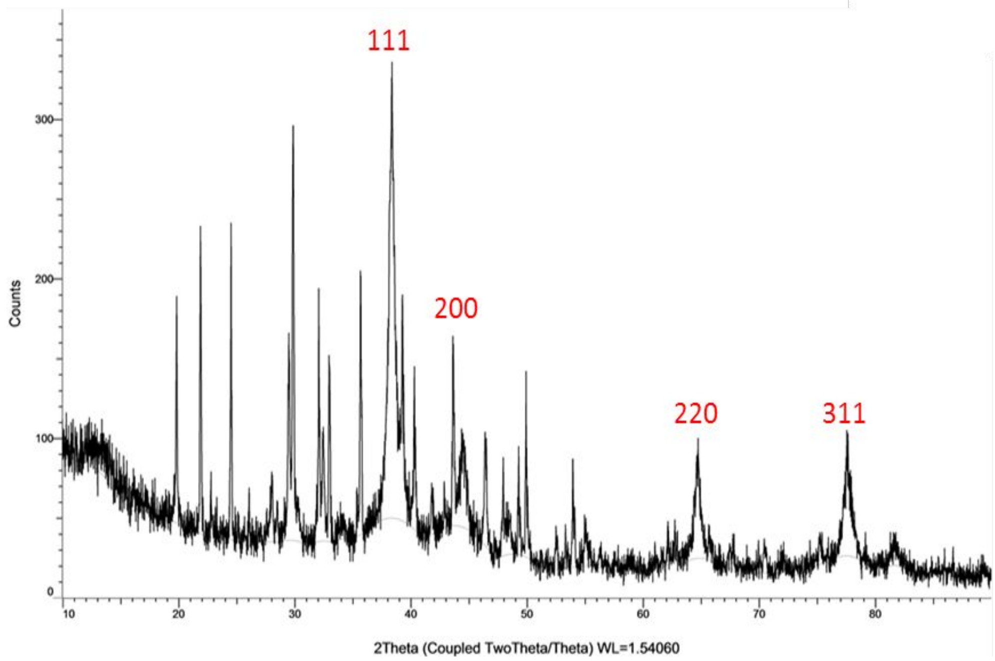

Figure S2. XRD models of AgNPs.

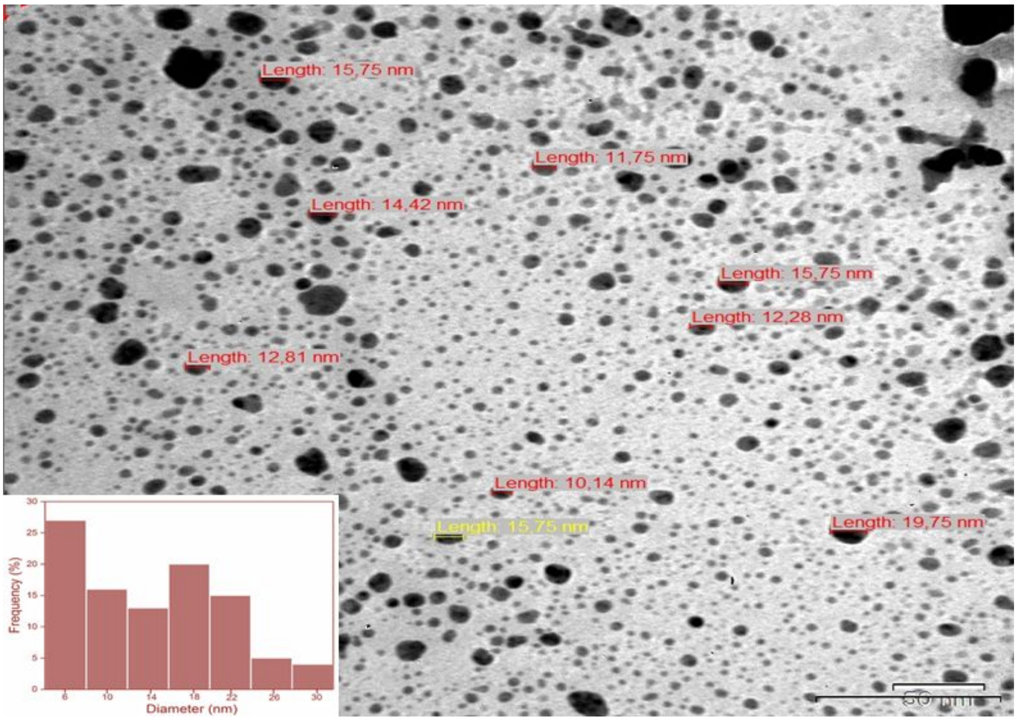

Figure S3. TEM images and histogram of AgNPs.

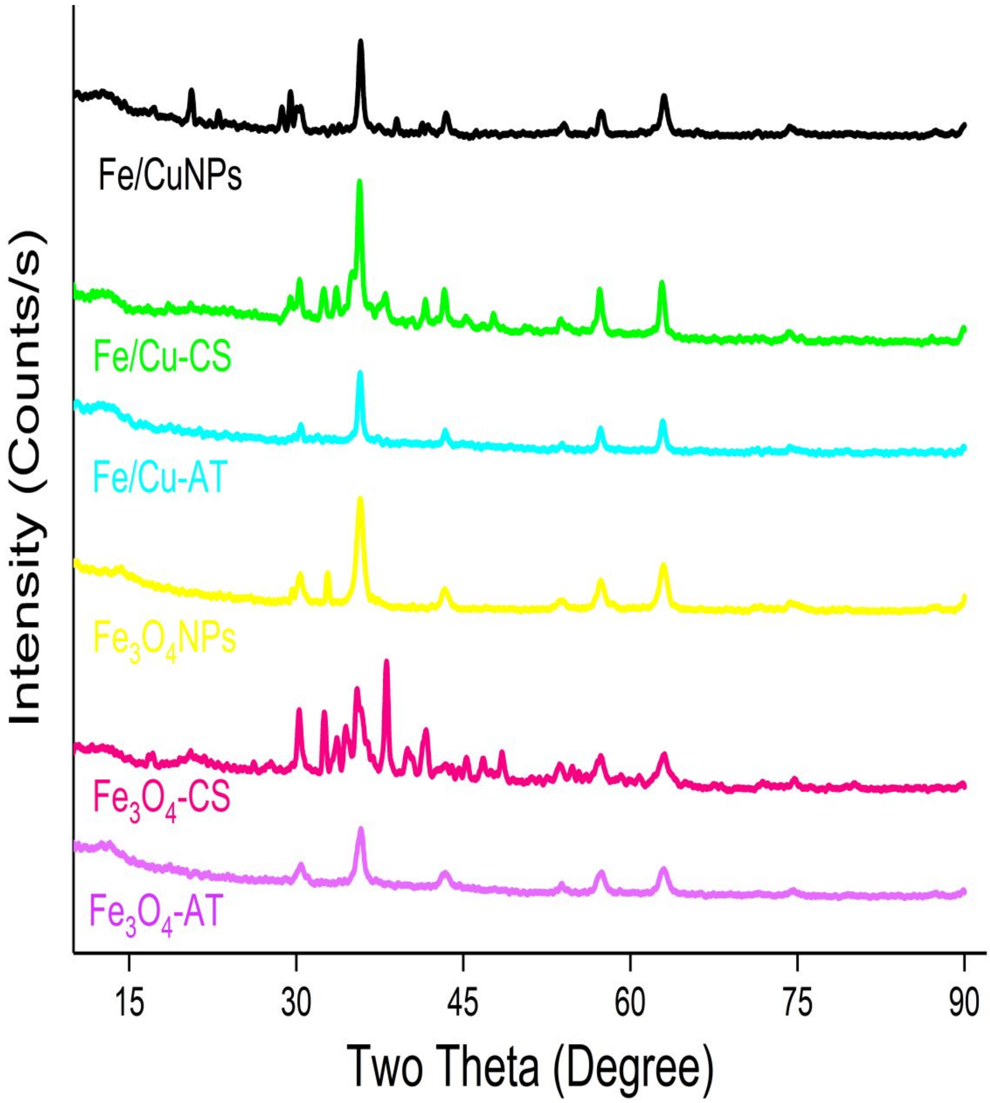

Figure S4. XRD models of nanoparticles/nanocomposites.

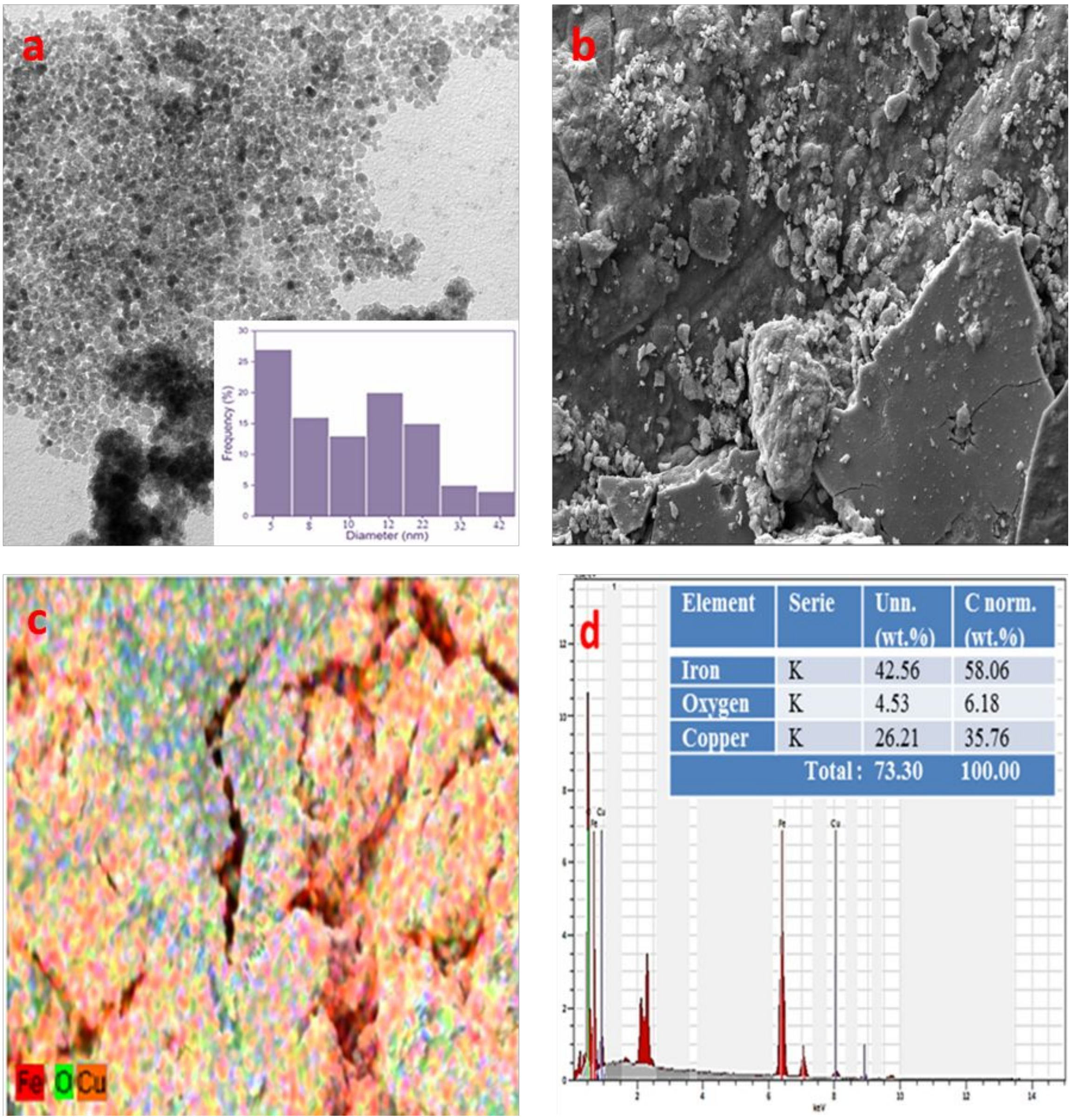

Figure S5. (a) TEM images and histograms of Fe/CuNPs (b) SEM images of Fe/CuNPs (c) Elemental mapping of Fe/CuNPs and (d) EDX analysis of Fe/CuNPs.

References:

(1) Şahin, M.; Arslan, Y.; Tomul, F.; Yıldırım, B.; Genç, H. Green synthesis of silver nanoparticles using Lathyrus brachypterus extract for efficient catalytic reduction of methylene blue, methyl orange, methyl red and investigation of a kinetic model. *React Kinet Mech Catal.* **2022,** 135, 3303-3315.

(2) Şahin, M.; Arslan, Y.; Tomul, F. Removal of naproxen and diclofenac using magnetic nanoparticles/nanocomposites. *Res Chem Intermed.* **2022,** 48, 5209-5226.
